# Review on Smart Electro-Clothing Systems (SeCSs)

**DOI:** 10.3390/s20030587

**Published:** 2020-01-21

**Authors:** Abu Sadat Muhammad Sayem, Siew Hon Teay, Hasan Shahariar, Paula Luise Fink, Alhussein Albarbar

**Affiliations:** 1 School of Fashion, Manchester Metropolitan University, Manchester M15 6BG, UK; P.Fink@mmu.ac.uk; 2 School of Engineering, Manchester Metropolitan University, Manchester M15 6BH, UK; S.Teay@mmu.ac.uk (S.H.T.); A.Albarbar@mmu.ac.uk (A.A.); 3 Department of Textile Engineering, Chemistry & Science, North Carolina State University, Raleigh, NC 27606, USA; hshahar@ncsu.edu

**Keywords:** smart garments, e-textiles, biosignals, sensors, dry electrode, signal-to-noise ratio (SNR), Internet of Things (IoT), knitted fabrics

## Abstract

This review paper presents an overview of the smart electro-clothing systems (SeCSs) targeted at health monitoring, sports benefits, fitness tracking, and social activities. Technical features of the available SeCSs, covering both textile and electronic components, are thoroughly discussed and their applications in the industry and research purposes are highlighted. In addition, it also presents the developments in the associated areas of wearable sensor systems and textile-based dry sensors. As became evident during the literature research, such a review on SeCSs covering all relevant issues has not been presented before. This paper will be particularly helpful for new generation researchers who are and will be investigating the design, development, function, and comforts of the sensor integrated clothing materials.

## 1. Introduction

The electrical, chemical, and mechanical activities that take place in the human body during any biological event, such as beating of the heart and contraction of muscles, produce different biomedical signals [1]. On the basis of the physiological origins of these biosignals, they can be grouped as bioelectrical, biomagnetic, biochemical, biomechanical, bioacoustics, bio-optical, and biothermal signals. They can be further classified based on their nature of existence, that is, permanent or induced biosignals [2]. Permanent signals exist at all times within the body and are generated without any artificial trigger, impact, or excitation from outside of the body, for example, electrocardiogram (ECG) signal. Induced biosignals are artificially triggered, excited, or induced and they exist roughly for the duration of the excitation, for example, electroretinogram (ERG). Sensors that can sense biosignals or biopotentials can be categorised as physical, electrical, or chemical depending on their specific applications [1]. Different kinds of specialised electrodes are used for capturing biosignals. These electrodes could be either non-invasive (placed on skin surface) or invasive (e.g., microelectrodes or wire electrodes). Adding electrodes and sensors onto textiles and garments is a non-evasive way of capturing and measuring biosignals.

**Figure 1:**
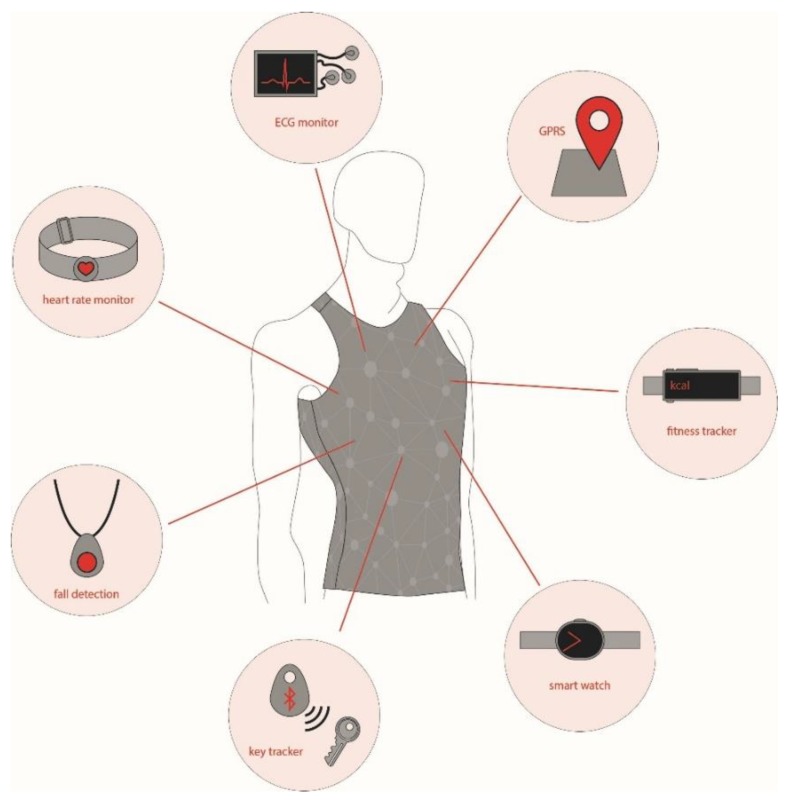
Concept of Wearable 2.0. ECG, electrocardiogram.

Thanks to the advancement of technology in producing microelectromechanical systems (MEMSs), wearable electronics have become very common consumables on the market nowadays. Wrist-worn wearable devices (smart watches and fitness trackers) experienced a growth of 18% and 7% in the United Kindom during the period of 2016–2017 and 2017–2018, respectively [3]. With the advent of conductive threads, textile structures either woven or knitted from conductive yarns, and conductive print-inks including those from graphene, it is now possible to produce or integrate light-weight sensors onto textiles to monitor health, fitness, and performance in a non-clinical environment, in daily-life, and in sport-training conditions [4,5,6,7]. An overview of the recent developments in wearable sensors for remote health monitoring is presented by Majumder et al. [8], while the smart sensors and fusion systems for sports and biomedical applications are reviewed by Mendes Jr. et al. [9]. In some cases, smart sensors are worn directly on the body using belts, straps, and adhesives; and in some cases, they are integrated or pocketed within textiles. The concept of Wearable 2.0 [10] envisages a full integration of wearable electronics within clothing, as presented in the Figure 1. Traditionally, such systems are known as smart garments, e-textiles, and e-garments. In the literature, they have also been mentioned as the IoT (Internet of Things) smart garments system [11]. For the ease of understanding across all disciplines, we have referred to them as smart electro-clothing systems (SeCSs) in this review.

A good number of SeCSs have emerged onto the market. This paper reviews the state-of-the-art development in design, construction, functionality, and application of such systems. As far as is known, such a review on SeCSs covering these relevant issues has not been presented before. However, it is important for researchers and product developers to have a complete review of those before initiating new research and attempting new product development in this and associated fields. 

## 2. Types of SeCSs Based on Applications

**Figure 2:**
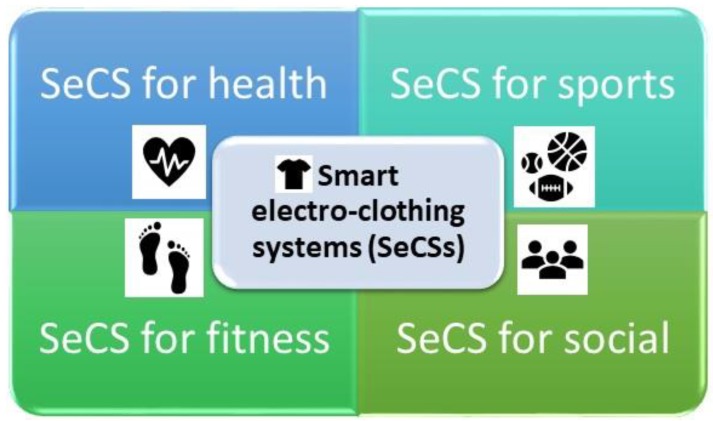
Classification of SeCSs.

On the basis of their areas of application, SeCSs can be classified into the following four groups (Figure 2):

(1) SeCS for health;
(2) SeCS for sports;
(3) SeCS for fitness;
(4) SeCS for social.

The textile-based systems that can measure biosignals, for example, ECG, body temperature, and so on, can be used for detecting and monitoring medical conditions, and can support recovery and rehabilitation; those are promoted by their suppliers for medical applications are identified as ‘SeCSs for health’ in this paper. The systems, which are promoted by their suppliers for sport applications, including monitoring players’ and athletes’ physical conditions and performance, and helping players/athletes and their coaches in training and coaching, are considered as ‘SeCSs for sports’. The systems that help general consumers with their daily fitness activities, such as walking, jogging, running, doing yoga, and physical exercises, are reported as ‘SeCSs for fitness’ in this review. The systems that do not fall into any of the above-mentioned categories, but facilitate users’ social activities such as communication, entertainment, and leisure activities, are identified as ‘SeCSs for social’.

## 3. Design Criteria for SeCSs

Most of the clothing materials we wear day-to-day are made of flexible textile fabrics that are either woven or knitted out of linear textile structures known as threads and yarns consisting of one of more types of natural or man-made fibres [12]. The basic characteristics of textile materials are that they are soft and flexible materials that are able to drape the curves of our body nicely. The fundamental requirements for them are their capability to ensure physiological (or thermo-physiological), sensorial (or tactile), and psychological of wearer [12]. At the same time, they should be washable to meet the users’ desire for reuse. However, these requirements are negatively interfered with when hard and non-washable electronics and electric materials are assembled with textile materials. This is the biggest challenge in designing SeCSs. Until now, it is not technically possible to have fully flexible and washable electronics components that can be assembled seamlessly with textile materials. Therefore, the trend is to make MEMSs in such a way that they minimally interfere with wearers’ comfort and can be detached from the clothing component before washing. The next challenge of designing SeCSs is to place appropriate electrodes and sensors in appropriate places on clothing so that they can come into sufficient contact with wearers’ body parts to be able to sense the targeted biosignals as purely as possible. For example, ECG sensors are usually positioned at the chest and ribs area and a blood oxygen sensor is placed at the triceps of the left or right muscles [10,13]. In addition to accurate positioning, it is also important to ensure no or minimal movement of them to avoid any noise in signals, also known as the motion artefact. 

## 4. System Architecture

**Figure 3:**
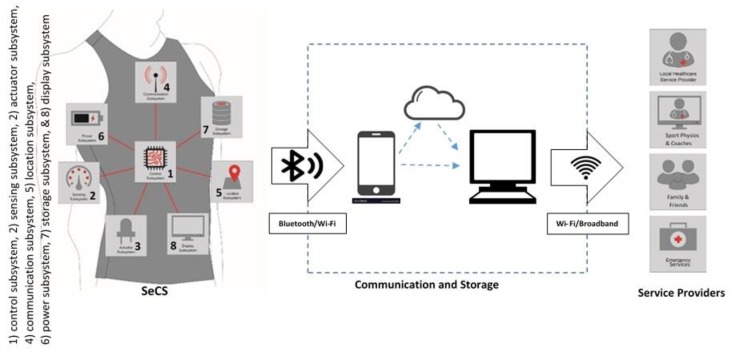
General system architecture of a SeCS.

Every SeCS consists of both hardware and software items. The generic architecture of SeCS is presented in the Figure 3. The system architecture generally includes eight working subsystems and two supporting subsystems. The common working subsystems included in a SeCS are as follows: (1) control subsystem, (2) sensing subsystem, (3) actuator subsystem, (4) communication subsystem, (5) location subsystem, (6) power subsystem, (7) storage subsystem, and (8) display subsystem [11]. Two supporting subsystems included in SeCSs are interconnection and software subsystems. Most of the hardware items in a SeCS construction, such as the control subsystem, certain types of sensing and actuator subsystems, location subsystem, power subsystem, storage subsystem, and display subsystem, are electronics and non-textile materials. Except the display subsystems, in most of the cases, the rest of these subsystems are accumulated within an electronic board in as miniaturised a form as possible to finally connect to textile components [13,14].

Different sensing units that potentially form sensing subsystem of an SeCS can be motion, gesture, and position sensors, temperature and other bio-vital sensors, location sensor, interaction and environmental sensors, and sensors for detecting surrounding objects [4,5,6,7,8,9,10,11,12,13,14]. The common sensors for motion, gesture, and positions are accelerometer, magnetometer, and gyroscope. A combined package of accelerometer, magnetometer, and gyroscope is common in use owing to their volatile application and prices. This combination is termed as a nine-axis intertial motion unit (IMU) sensor. STMicroelectronic’s LSM9DS1 and Bosch’s BMF055 are two examples of such IMU sensors. Potential bio-vital sensors that may be integrated within a SeCS are for sensing heart rate, respiration rate, blood pressure, pulse oxygenation, glucose levels, and galvanic skin response, or electromygraphy (EMG), ECG, electroencephalogram (EEG), and so on [8,9,10,11,12,13,14]. Actuators for SeCSs include visual indicators, sound, movement and vibration, and heating and cooling [8,9,10,11,12,13,14].

## 5. Construction of SeCS

**Figure 4:**
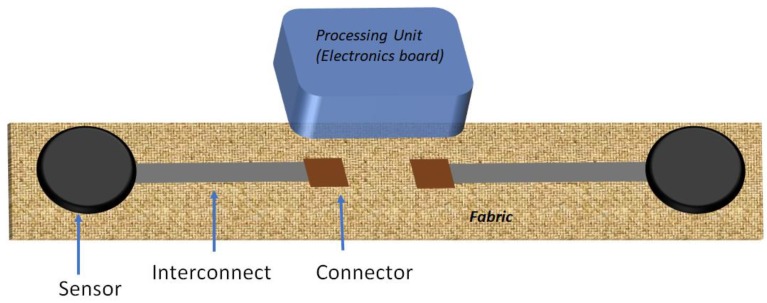
Interaction between textile-based and non-textile-based subsystems of a SeCS.

Textile fabrics work as the basic platform for integrating different subsystems in and on them to construct a SeCS. Figure 4 represents the interaction between different textile-based and non-textile-based subsystems of a SeCS. The interconnects transfer power and biosignal between the sensor point and the data processing unit (electronic board). The sensor units are linked to a rigid electronics board by connectors. The quality and reliability of the sensor integrated into smart garments are fundamentally dependent on these constituent components of SeCSs. Any failure of any of these three components will cause the device to malfunction. Any sensor needs to be highly and selectively sensitive to biopotential (e.g., ECG, EMG, and EEG) or other targeted markers. The interconnect and the connector should have very low resistance and high durability, so that the resistance of these components does not change after repeated mechanical agitation during washing, bending, and stretching of the device. Traditionally, metallic wires and components act as an interconnector for electric items; however, textile-based interconnects are becoming popular for SeCSs to comply with the requirements of wearability and washability.

As mentioned before, the connector connects the soft textile electronic parts with the rigid electronics board. Snap buttons, low-melting temperature soldering, and conductive epoxy bonding are the common ways to connect the hardware piece onto the textile. The limitation such a joint is that any stress concentration on the connector when the fabric is strained may cause the joint to crack between the soft and hard segments of the device. Unfortunately, there has not been much research conducted to solve the connector problem for the electronic-textile industry. It is shown that utilisation of non-conductive epoxy-glue encapsulation on top of the conductive and brittle connectors improves the durability and the lifetime of the device [15,16].

### 5.1. Textile Fabrics

For SeCSs, the electronic components are either attached or developed directly on textiles. This section reviews the variety of textile materials available for potential use as base materials for constructing SeCSs. Textile materials come in any of the following geometrical structures and physical appearances: (a) fibre (including filament), (b) yarn and thread, (c) fabric, and (d) assembled product (clothing and non-clothing). Fibre is a hair-like pliable material, which is considered as the basic and starting unit of a textile material. Yarn, an intermediate product between fibre and fabric, is made of fibres twisted together [12]. A fabric is either made by interlacing of two sets of yarns or by interlocking of loops formed by one set of yarn. The former process is known as weaving and the latter one is known as knitting, from which the resultant materials got their names as woven and knitted fabrics, respectively.

Woven fabrics commonly come in any of the following design groups: plain, twill, and satin fabrics, where each of them may have different derivatives. Owing to difference in weave design, the fabrics of these three classes will have different textures and different properties, for example, tensile strength, even if they are made from exactly the same type of yarns. The machine used for weaving is commonly known as a loom, and there are varieties of looms available such as shuttle looms including tappet, dobby, and jacquard looms, and shuttle-less looms including projectile, rapier, water-jet, air jet, and so on [17]. 

The varieties of knitted fabrics produced commercially are either weft or warp knitted fabrics. A weft-knitting machine can produce three basic knit designs such as plain, rib, and purl [18], and the fabrics produced are known by the design they contain. Each of the basic weft knit designs may have many derivatives within each design group. Warp knitted fabrics can be of seven different types: namely, tricot, raschel, ketten raschel, milanese, simplex, crochet, and weft-insertion warp [19]. 

There is another category of fabric, which is made directly from fibres using different bonding technologies including chemical, mechanical, and thermal bonding technology, among other. They are known as non-woven and can be classified into three groups based on the techniques used to lay the fibres together, such as drylaid, wetlaid, and polymer-laid (or spunmelt) [20]. This type of material is commonly used as padding and filler within clothing.

Clothing materials made for our use or sometimes for animal use are assembled products out of mostly fabrics combined with threads and minor non-textiles materials such as button, zippers, hook and loop fastener, rivet, and so on.

### 5.2. Textile-Based Sensors and Electrodes

Although several types of sensors are incorporated within SeCSs, only a few of them are actually developed on textile surfaces directly, such as ECG, EMG, and temperature sensors. As ECG and EMG sensors detect electrical signals from the skin surface, the fundamental principle of developing such sensors directly on textiles is to make the textile surface conductive. Traditionally, disposable wet electrodes containing conductive silver/silver–chloride (Ag/AgCl) ink, printed on an adhesive paper and coated with ionically conductive gel (typically hydrogel), are used to measure the ECG signal from the heart activity. The ionic gel creates the ionic bridge between the body and the electrodes and lowers the skin to electrode impedance. Additionally, the AgCl salt in the conductive ink also helps to maintain the ionic bridge network between the skin and the electrode. The skin alike soft gel material can enhance the adhesion of the electrodes with the skin, and thus the minimise motion artifact of the signal. However, these sticky sensors can cause discomfort and noticeable body rash if used for a long time [21]. Therefore, textile-based dry electrodes are heavily studied as an alternative to commercial wet electrode for long time monitoring of vital signs, even in the non-hospital condition.

Screen printing of conductive ink directly on substrates like, film, textile, and nonwoven materials is used as a simple and common technique to develop sensors and electrodes for measuring the electrical signal from the skin surface. Increasing the surface area of the electrode can potentially decrease the skin to electrode impedance and provide a reasonable signal with a comparable signal-to-noise-ratio (SNR) to commercial wet electrodes [22]. Ag/AgCl ink is dominantly used for screen printing dry electrode to enhance the ionic conductivity and lower the skin to electrode impedance, although this requires the generation of sweat on the skin. Dry electrodes show promise in the literature as durable sensor electrodes for long time monitoring; however, the signal quality deteriorates drastically when the wearer is in active mode such as walking or running, as dry electrodes cannot create a good adhesion on the skin like wet electrodes. Integrating dry electrodes at the strategic locations (where the body muscles do not move much during active modes) in compression garments enhances the signal quality [23]. Other than conductive Ag ink and Ag/AgCl ink, functional materials including carbon [24] conductive polymers such as PEDOT/PSS [Poly(3,4-ethylenedioxythiophene)-poly(styrenesulfonate)] [25] are used to measure signals like ECG. These active materials are directly screen printed, inkjet-printed [26], or dip-coated [25] on textile to develop wearable sensor electrodes.

Electrically conductive yarns can be integrated in the fabric structure to develop a conductive patch that can also be as used textile sensors to measure human physiological vital signs. The whole garments knitting technology can enable developing a garment with a diverse design without the need for cutting and sewing. This platform technology can integrate a conductive yarn and knit sensor patched in the designated location of a garment. Knitted sensors improve wearers’ comfort owing to their breathability, however, they require high compression to detect a quality signal [27]. Additionally, technology like embroidery of conductive yarn on a textile can create dense conductive patterns on a textile surface and create a cushiony structure to impart compression on the sensor location to improve signal quality [28]. 

Other than these common materials and manufacturing processes, recent development of ionically conductive inkjet printable materials show great promise for manufacturing biosensors on different substrates including textiles. The combination of inkjet-printed conductive polymer electrodes and ionically conductive materials on top as a coating lowers the skin to electrode impedance and improves the SNR of the signal [26]. It is already mentioned that the ionically conductive and tacky hydrogel material is used in almost all commercial electrodes to lower the skin to electrode impedance, improve ionic conductivity and lower the motion artifact. However, these gel materials are not durable and dry out over the time. Recent development of durable, tough and conductive hydrogel opens the new avenue of electronics called “ionotronics”. These materials already show a superior result in sensing bio-signals (including ECG) from the human skin for a long period of time [29]. However, the multiple manufacturing steps and integration challenge of this material with other soft materials such as textiles are yet to be progressed for commercial applications.

The concept of creating a secondary skin-like material that act as sensors and feels like skin poses a unique idea for building biosensors. The development of gecko-like dry adhesive with conductive functionality shows promising results to monitor bio-signals from skin in real time during heavy action periods of the wearer. The literature shows the development of conductive soft silicone materials with micro-patterned top surface can adhere to the human skin, simulating the gecko feature [30]. The sensor shows significant improvement of the motion artefact, which is a great challenge for the class of dry electrodes.

### 5.3. Textile-Based Interconnects

The interconnects are developed on textile using different techniques, such as by screen printing of conductive thick paste directly on the fabric [31] or on a transferable thermoplastic film [32], stitching or embroidery of conductive yarns, direct knitting or weaving of the conductive yarns as an interconnect pattern, and so on [33,34]. Screen printing of conductive thick paste ink has been a common practice to print interconnect on non-stretch plastic film for different printed electronics applications. However, the screen-printed metal film layer delaminates or cracks when the interconnect is subjected to the stretching owing to the mismatch of the mechanical properties of the printed film and the substrates. [32,35]. On the other hand, interconnects stitched, knitted, or woven with metal filament integrated or electroless plated metallised conductive yarns are more durable during stretching [36]. Recent progress in the inkjet printing of particle-free reactive metal–salt solution directly on textile fabric can metallize the yarn at the molecular level. Such a method could potentially solve the problem of durability, patternability, and scalability of textile interconnects [36].

## 6. Available SeCSs on the Market

**Figure 5:**
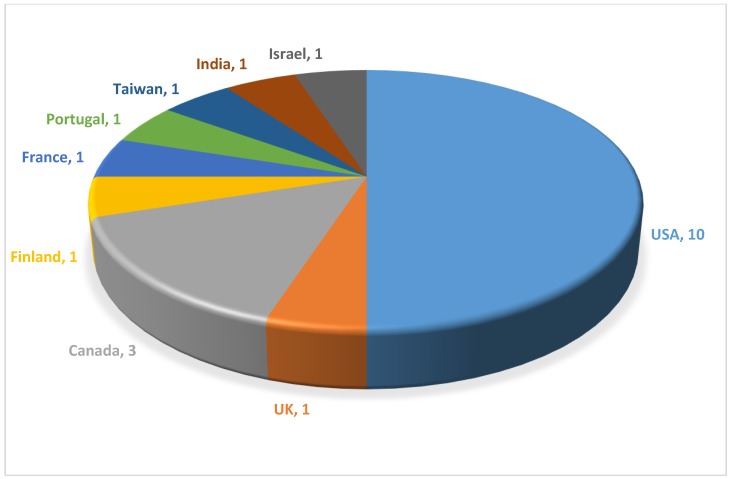
Distribution of SeCS suppliers worldwide.

Table 1 provides a list of SeCSs that are being offered on the market or in the offing and their suppliers. Among the twenty-two companies [37,38,39,40,41,42,43,44,45,46,47,48,49,50,51,52,53,54,55,56,57,58,59,60] listed there, the USA and Canada dominate with the most number of suppliers offering SeCSs (see Figure 5). In contrast, only few companies are active on the European market. Interestingly, no Chinese company has been identified as being active on the global market. It is also noticeable from Table 1 that more than half of the suppliers are offering health monitoring SeCSs, while SeCSs for sports are the next leading product category. Most of the companies offer complete garment solutions to the consumers and only one company supplies compression sleeves. While the most of the companies target adults, only a few focus on babies with their product offers. Pricewise, the available SeCSs fall within the price range of luxury products except those that come from India.

### 6.1. SeCS for Health

Twelve companies have been identified as the suppliers of SeCSs that can capture biosignals from the human body and their proprietary software systems can analyse those signals to report the well-being of the wearers. Table 2 and Table 3 summarise the features of these products and the following subsections discuss them briefly. Out of these companies, as can be seen in Table 2, OMsignal, Myant, and Smartlife are offering technology rather than any readymade product to the consumers directly. The common features of the SeCSs of this category are the use of knitted fabrics as the base clothing material upon which the electronic components are attached and integrated. The products from Neopanda and Mimo are focused dedicatedly to new-borns and babies, and the rest are for grown up consumers.

#### 6.1.1. Vital Jacket from Biodevices

Vital Jacket or VJ^®^ system from Biodevices S.A. (Portugal) [37] is claimed to be the first SeCS certified as medical device by the European Union’s (EU) medical device directives (MDD)—93/42/EEC [64] for collecting ECG data [37]. The hardware system consists of a t-shirt or a vest as a carrier of conductive pathway (covered electric wire), digital recorder, SD card, battery charger, and disposable electrode. For capturing biosignals, one or more electrodes need to first be placed on the recommended areas of wearer’s body, and the t-shirt is then donned to facilitate the electrodes to be connected with its cables. The recording device is connected inside of a pocket located at the level of the side-waist. The system allows to collect ECG data from a wearer using commercial wet-electrodes for a long period of time, transmits remotely, and stores all data for posterior analysis. It can measure a patient’s movement using a three-axis accelerometer. Vital Jacket is available in two versions (with 1 or 5 leads) for babies, children, and adults, and both can perform an ambulatory ECG. It has an analysis software specific for rhythm alterations, which can only be read by a health professional. Experimental applications of VJ include stress detection in bus drivers [65] and firefighters [66] through analysis of ECG data and heart rate variability (HRV), identification of physiological responses to stress in musicians [67] through monitoring heart rate as beats per minute (bpm), and studying stress and fatigue of first responders through ECG and continuous blood pressure monitoring in laboratory condition [68].

#### 6.1.2. Hexoskin

Hexoskin from Montreal, QC, Canada is a wearable health monitoring system that includes ECG electrodes integrated into clothing and an e-module including breathing and movement sensors [41]. The system can measure heart rate (HR), heart rate variability (HRV) and heart rate recovery (HR2), breathing rate and volume, movement, step count, cadence, stride, activity level, calories burned, and sleep quality. According to the supplier, the system has found research application in the areas of cardiac, respiratory, and activity analysis (such a steps, cadence and calories, stress, cognitive, and sleep). Abdallah et al. [69] applied the Hexoskin biometric vest to measure ventilation (VE), tidal volume (VT), breathing frequency (Bf), inspiratory capacity (IC), and inspiratory reserve volume (IRV) of a small cohort of adults with chronic obstructive pulmonary disease (COPD) at rest and during exercise; and found them to be valid when compared against the data collected by a pneumotachograph (Ptach). Al-Sayed et al. [70] compared the heart rate monitoring capacity of the Hexoskin biometric shirt and Polar H7 heart rate sensor through a study involving twelve volunteers and reported no significant difference between the two systems. Banerjee et al. [71] employed the Hexoskin vest to estimate physiological measures such as heart rate, breathing rate, lung volume, step count, and activity level of thirty-one participants aged 65 and older and compared the collected data against the clinically accepted gold standard values. They concluded that heart rate, breathing rate, and step count collected by Hexoskin showed a strong correlation against the gold standard measures, but lung volume and activity level measures did not.

#### 6.1.3. OM Signal

OM signal from Canada offers SeCS with embedded ECG, respiration, and physical activity sensors [39]. The system contains a printed ECG sensor on the inner surface of the clothing. The e-module is attached on the left side under the chest area of clothing (shirt, camisole, and bra). This records the consumer’s biosignals and streams them wirelessly and in real time to the consumer’s smartphone via Bluetooth. The data are also automatically sent to the cloud platform of the Internet, where it can be further analysed using advanced algorithms and artificial intelligence (AI). Porbabee [72,73] applied this system in ECG-based human identification and mental stress prediction using heart rate variability. However, the system is yet to be offered commercially.

#### 6.1.4. Emglare

Emglare from California, USA offers a SeCS with fully integrated heart rate and EC sensors, non-detachable battery for wireless charging, and a Bluetooth antenna [40]. The intelligently designed vest hosts two ECG sensors and one heart rate monitor at the chest area in the front and an automatic power switch near the left armhole. The Bluetooth antenna, battery, and wireless charging component are hidden at the centre back of the clothing. Once a consumer wears the vest and turns on the Emglare mobile app, the smart vest starts sending heart rate and ECG data automatically to the app. The system stores health statistics on a daily and weekly basis, which can be shared with others. The application automatically sends a notification if the heart rate is higher than usual and can inform connected doctors, relatives, or friends about it. Although it looks very smart in design and activity, the system is yet to reach consumers’ hands. However, the company is accepting online pre-orders now.

#### 6.1.5. Master Caution^®^ from Healthwatch

Master Caution^®^ from Healthwatch Technologies, Israel offers a 12-lead ECG monitoring garment with Food and Drug Administration (FDA)-clearance and European Union’s CE-approval [41]. The system can monitor heart activity, respiration, fall detection, movement, and temperature. The design is based on wearable textile-electrodes and heart-sensors and contains digital health diagnostic services including mobile cardiac telemetry, patient monitoring tele-health services, and other services that allow for in-home medical services. Master Caution^®^ continuous monitoring solutions assist clinicians in remotely monitoring their elderly or bed-ridden patients. It can alert to cardiac events such as ischemia and arrhythmias in near real-time, using the automatic analysis (AI) system, and thus securing personal health around-the-clock for improved patient safety. According to the supplier, the offered garment is machine washable, with at least 50 washing cycles, and is available in a full size range for men and for women.

#### 6.1.6. Siren Diabetic Socks

Siren from San Francisco offers socks with embedded temperature sensors that can help detect foot ulcers early in diabetic patients [42]. It uses temperature micro-sensors integrated into textiles that can detect changes in temperature at the bottom of the feet. A small tag attached to the sock reads this temperature gradient data and wirelessly transmits it via Bluetooth to a specific app. A study by Armstrong et al. [74] shows that self-monitoring of foot temperature may reduce the risk of ulceration in diabetic patients.

#### 6.1.7. Neopenda Baby Hat

The New York based company Neopenda (New York, NY, USA) aims to fight the sudden infant death syndrome (SIDS) in developing countries, namely Uganda. It offers a baby monitoring hat that makes it possible for nurses to monitor several infants continuously and simultaneously, thus reducing newborn mortality [43,44]. The baby hat is embedded with an e-module at the front and is able to measure temperature, heart rate, respiratory rate, and blood oxygen saturation of the infants. The device can transfer vital signs to a central monitoring system via a Bluetooth transmitter. The system is designed to monitor up to twenty-four babies through one monitor.

#### 6.1.8. Mimo from Rest Devices

Similar to Neopenda, Mimo from Rest Devices (USA) is a baby breathing and activity monitoring kit that includes machine washable kimonos of a specific size (0–3, 3–6, and 6–12 months), one Lilypad (charging & WiFi base station), one low-power Bluetooth transmitter called turtle, and charging and power cables [48]. The e-module is in the shape of a green turtle and snaps onto the front of the onesie, and can monitor the baby’s breathing, body position, sleep activity, and skin temperature. Mimo data strips pick up subtle movements in baby’s breathing and activity and transmit those to the Lilypad, which sits near the baby while plugged into a wall. The Lilypad picks up baby’s coos and cries through an embedded microphone, and sends that live audio, along with all other data, securely to a server and then straight to parents’ smart devices, where they can see, in real-time, how their little ones are doing.

#### 6.1.9. Bioman+ from AiQ

The Taiwanese company AiQ’s smart clothing offers a variety of smart garment, under the general name Bioman+, with an integrated 1–3-lead ECG monitoring system for health monitoring of patients, elderly people, and sportspersons [47]. It is an upper body garment solution that consists of conductive fibre-based textile electrodes for the acquisition of the electric activity of human body and conductive thread to carry the electric signals to the processing and transmission module that is snapped onto the garment. It is available in several styles—vests, t-shirts, and sportbras—with five different types of electrode structures suitable for different user scenarios and three fabric variants with different levels of compression. The company claims to have used stainless steel fibres, yarns, and threads, omitting the need for an additional copper or silver coating, to simplify manufacturing [75].

#### 6.1.10. Skiin from Myant Inc.

The Canadian company Myant Inc. offers smart fabrics under the brand “Skiin” that are claimed to be comfortable and washable, and able to monitor ECG, HRV, breathing patterns, stress levels, sleep quality, steps, distance, calories burned, active minutes, and stationary time all day and night. For female consumers, it can also identify the fertility window by monitoring the changes in skin temperature and resting HRV to maximise the chances of getting pregnant [48]. The company has presented the design of underwear in classic cuts in varying fit for both men and women. Each undergarment has a slit in the waistband where the smart device can be inserted to track the health of the wearer [76]. The device can be charged wirelessly. The company is offering smart fabrics and smart solution for retailers; therefore, the final product is yet to be available commercially.

#### 6.1.11. Neuronaute^®^ from BioSerenity

The French company BioSerenity offers a SeCS called Neuronaute^®^ for diagnosis and monitoring of patients with epilepsy in their own home [50,61]. The system consists of a smart t-shirt and a smart cap containing EEG, ECG, and EMG sensors and a nine-axis accelerometer. This top and cap outfit can detect electrical activity from the brain, heart, and muscles of its wearer and send it to a smart phone or to doctors via the Cloud [62,63]. The system obtained CE marking in 2016 after a six-month trial at the Brain and Spine Institute at the Pitié-Salpêtrière Hospital in Paris [53,64].

#### 6.1.12. Others

The British company Smartlife offers a textile sensor technology that can be integrated into comfortable active wear [49]. The company claims their device, called the Brain, to be small and discrete, allowing communication with third party apps. The textile sensors and smart device offered by them are claimed to be able to monitor ECG signals, impedance pneumography, impedance plethysmography, surface electromyogram, and accelerometry for 12 h.

The American company Sensoria offers SeCSs that can be of help for people suffering from gait impairments, short stride lengths, and slow walking speeds. The Sensoria^®^Walk app works in conjunction with an electronic anklet and textile sensor infused smart socks to help its wearer set goals, and track daily activities including steps, cadence, and distance during rehabilitation after a stroke or post-surgery, with the ultimate goal of speeding up overall recovery time. As reported by Gaibizzi et al. [77], the Sensoria smart t-shirt could potentially be a promising candidate component, which is compatible with the Heart Sentinel™ smartphone app, to build a system for detecting and alerting cardiac arrest caused by life-threatening arrhythmias such as ventricular fibrillation (VF) during outdoor sports. A study by D’Addio et al. [78] on posturographic assessment with a small group of patients with Parkinson’s disease identified Sensoria fitness e-textile socks as a low-cost alternative to evaluate variations in centre of pressure (CoP) signal when compared with the gold standard stabilometric Zebris platform (ZP).

### 6.2. SeCS for Sports

Five suppliers of SeCS were found to be active in the sports industry (see Table 4 and Table 5). Except Komodotec, all others offer clothing items for sportspersons. Komodotec offers a compression sleeve for the arm, which has an e-module encaged into it. Again, knitted fabric is the common feature of the textile components of these products.

#### 6.2.1. Athos

Athos system from Mad Apparel Inc. (USA) includes a compression shirt and a detachable e-module, which offers real-time biometric tracking, including muscle activity, heart rate, calorie expenditure, and active time versus rest time [51]. It tracks exertion of the major upper-body muscle groups: pecs, biceps, triceps, deltoids, lats, and traps. When snapped on the Athos apparel, its e-module can collect and analyse data from the garment’s sensors and delivers those data to the user’s mobile app via Bluetooth. The proprietary software can display which muscles are firing and how much they are being exerted; shows the distribution of work by muscle group, from left to right, to detect if the user is overworking or compensating as a result of poor form; and helps understand how muscles are contributing to the movement. It is reported by the supplier that athletes from different professional league in the USA including the Philadelphia Phillies (MLB), LA Clippers (NBA), FC Dallas (MLS), and Ohio State (Collegiate Division 1) use this system for training purposes. Lynn et al. [82] studied surface electromygraphy (sEMG) measurements from twelve healthy subjects taken by Athos compression garments with built-in EMG electrodes and research grade Biopac bipolar Electrodes (Biopac Systems Inc., CA, USA). Their findings showed no significant differences between normalized EMG amplitude or in strength of the relationship between sEMG and torque output between Athos and Biopac. 

#### 6.2.2. Zephyr from Medtronic

Zephyr™ performance system from Medtronic (USA) [52] is a SeCS designed to support training of athletes, military, and first responders. The system can read six parameters (ECG, respiration, estimated core body temperature, accelerometry, time, and location) of its wearers and can process them to report twenty one biometrics (heart rate, breathing rate, heart variability, HR confidence, estimated core temperature, impact, activity, posture, caloric burn, % heart rate, % heart rate anaerobic threshold (AT), accelerometry, physiological and mechanical intensity loads, training loads and intensity, jump, explosiveness, peak force, peak acceleration, GPS speed, GPS distance, and GPS elevation). The combination of these biometrics can yield nine biomarkers of a wearer, as follows: (1) fatigue (HR recovery), (2) readiness (HRV), (3) safety (maximum HR, core body temperature, location), (4) over-training and under training (intensity and load), (5) fitness improvement (VO_2_ max, HR @AT), (6) caloric expenditure and burn, (7) agility (accelometry, speed and distance), (8) athlete management (intensity and load), and (9) stress (HRV). Its sensor module known as BioModule™ can be worn via a compression shirt and sport bra or a strap. Nazari et al. [83], through a systematic review of literature, identified ten research studies focusing on the reliability and validity of heart rate measurements taken by the Zephyr device and concluded that the device displayed good agreement with the gold standard measurements.

#### 6.2.3. Polar Team Pro

Polar Team Pro offers a team-based solution for athletes and their trainers [53,81]. The performance tracking sensor embedded in the garments is able to track motion, heart rate, and location through GPS. All information gathered by the garment is then sent to a tab, allowing the coach of a sports team to evaluate all their players at once and from a distance of up to 200 m [84]. 

#### 6.2.4. Komodotec

Komodotec offers a smart compression sleeve, which can be paired with a separate sensor device to track heart rate, analyse sleep patterns, and provide full-time ECG monitoring [54]. The company claims the sleeve is easy to wear and does not interfere with everyday life. On the basis of heart rate variability, the sleeve can give information about the body’s reaction to alcohol or drugs, recovery status, the wearer’s stress level, and their reaction to food.

#### 6.2.5. Sensoria

The running system from Sensoria includes a smart t-shirt or sport bra and smart socks, and supports professional runners with their training and coaching [55]. The Sensoria Run mobile app allows them to tailor their goals and track their progress, and the Sensoria Virtual Coach literally monitors every step and provides actionable audio and video feedback during running. It can help professional runners to improve their running mechanics by telling them when they are in the correct and incorrect running positions.

### 6.3. SeCS for Fitness

Although there are several wrist-worn wearable systems that support fitness activities and tracking are available on the market, only a handful of SeCSs are there to serve this sector, as can be seen in Table 6 and Table 7. All of them are based on knitted platforms and the product range covers T-shirt, vest, sports bra, yoga pants, and socks.

#### 6.3.1. Sensoria Socks

In addition to the smart shirts described in Section 6.2, Sensoria offers smart socks with integrated textile pressure sensor technology [58]; when paired with a Bluetooth enabled anklet, it can track the user’s steps, walking time, and distance on a daily basis. The accompanying application allows to set independent goals on each metric that a user wants to track. The anklet is detachable, while the socks are infused with proprietary textile sensors. This allows the socks to monitor not only step counting, speed, calories, altitude, and distance, but also cadence and foot landing technique, while exercising. 

#### 6.3.2. Wearable X

Wearable X, an Australian American company, offers leggings, branded as “Nadi X”, with knitted accelerometer and haptic feedback technology for yoga training. It can track the wearer’s goal, performance, and progression to help personalised yoga training in a real-time yoga session [56]. In conjunction with its electronics component that integrates battery and Bluetooth data transmitter, the yoga pants can generate gentle vibrations to guide the wearer with yoga poses and can act as a yoga coach when paired with the Nadi X iOS app.

#### 6.3.3. Supa

The brand Supa from USA offers a sports bra with integrated textile heart rate sensors [60]. The e-module, called SUPA reactor, can be attached to the sports bra and is then connected to a proprietary app (SUPA.AI). As this system is made for active wear, the smart device is water resistance and can track workouts by monitoring the heart rate of the wearer, similar to a sport monitoring chest belt. It is also supported by artificial intelligence within the application. 

#### 6.3.4. Syngal T-Shirt from Broadcast Wearables

This t-shirt by the Indian company Broadcast Wearables is being offered for the use during exercise and everyday life or in traffic [57]. The garment is able to track steps and floors climbed. It also provides how many calories are burnt and distance achieved during exercise. Additionally, the t-shirt can help the wearer navigate in traffic. The company claims that the t-shirt vibrates slightly on the wearer’s shoulders to indicate the direction to turn. Compared with other garments in this category, this t-shirt does not include a heart rate monitor.

### 6.4. SeCSs for Social

Other than health, sport, and fitness sectors, there are a few SeCSs that can assist in communication, entertainment, and leisure activities of their users. This category of SeCSs includes woven fabrics in addition to knitted ones as the base platform onto which to attach electronics.

#### 6.4.1. Trucker Jacket by Levi’s & Google

American companies Levi’s and Google jointly presented a smart jacket that facilitated a smooth commute for cyclist in big cities. With conductive yarns woven into the sleeve of the jacket, it works as an electronic platform. Digital connectivity is provided through the snap tag attached to the jacket’s cuff. The snap tag, which is positioned at the cuff of the left sleeve, can communicate with the wearer through light and haptic feedback. The companies claim that the battery of the tag lasts up to two weeks and can be charged using USB. It is also claimed that wearing the trucker jacket, consumers will be able to connect to their digital life instantly and effortlessly. With a lateral brush of the cuff, the wearer can handle calls and texts without handling the mobile device, as well as navigate and play, pause, and skip through their favourite music [78,79].

#### 6.4.2. Spinali Design

The French company Spinali Design offers different clothing ranges and swimsuits with embedded sensors for intelligent functions [60]. One of those functions is UV warning to the wearer via smartphone app to apply sunscreen. Its iOS/Android associated system “the Neviano UV Protection” comes with the functions like “weather”, “pics”, “suntanning tips”, and “sunscreen alert”. The application integrates a function called “Valentine” that alerts users’ partners when to apply sunscreen to the users while sunbathing. 

## 7. Research Gaps and Conclusions

It is evident from the Section 6 that only a few companies are offering SeCSs around the world. With the expansion of IoT application in various fields, it is expected that this number will grow gradually. However, there has been no study presented so far about consumers’ perceptions and demands of SeCSs. The trend in wearable technology is to have them embedded within clothing, known as Wearable 2.0, which also envisages being convenient, comfortable, washable, highly reliable, and durable. At present, wearable electronics are mounted on textiles, but not fully embedded into textiles. They are only washable at the moment when electronic components are removed from them. There has so far been no study reporting how the rigid electronic components influence consumers’ comfort perception. Another significant research problem is the energy sustainability and battery size, to move towards true Wearable 2.0. So far, only the sensor subsystem, out of eight subsystems of SeCSs, can be developed directly on textiles. The next step will be to attempt to develop other subsystems on textiles. The available SeCSs are only washable after detaching the electronics components from them. Developing a waterproof enclosure for e-components onto textiles is the prevailing challenge that needs to be addressed though research and development (R&D).

## Figures and Tables

**Table 1:** List of smart electro-clothing systems (SeCSs) available on the market.

#	Type	Base Product	Supplier	Origin	Price	Ref.
1	Health Monitors	T-shirt and Vest for adults, children, and babies,	Biodevices SA	Portugal	€ 650~750 (excl. software)	[37]
2		Shirt (Men’s, Women’s, and Junior’s)	Hexoskin	Canada	US$499	[38]
3		Shirt (Men’s and Women’s), Bra	OM Signal	Canada	-	[39]
4		Vest, port T-Shirt, Bra, and sport Bra	Emglare	USA	US$199	[40]
5		Vest	Healthwatch	Israel	-	[41]
6		Socks	Siren	USA	US$19.95/month	[42]
7		Baby hat	Neopanda	USA	US$75	[43,44]
8		Baby kimonos	Mimo	USA	US$199	[45,46]
9		T-shirt	AiQ	Taiwan	NTD 2980	[47]
10		Underwear	Myant	Canada	-	[48]
11		t-shirt, vest, or bra	Smartlife	UK	-	[49]
12		Shirt and cap	BioSerenity	France	-	[50]
13	Sports Training Aid	Shirt (Men’s) Shorts (Men’s), Leggings (Women’s)	Athos	USA	Men’s Shirt US$398, Leggings US$348	[51]
14		Compression T-shirt, Sports Bra, loose fit shirt (Men’s and Women’s)	Medtronic	USA	T-shirt US$199 Sport Bra US$155 Loose fit shirt US$173 (excl. e-module)	[52]
15		Sleeveless T-shirt	Polar Team Pro	Finland	-	[53]
16		Compression Sleeve	Komodotec	USA	US$144.95	[54]
17	Sports Training Aid& Fitness Tracker	T-shirt, Vest, Sports Bra, Socks with anklet	Sensoria	USA	T-shirt, Vest US$129~139, Bra US$119, Socks + anklet US$199	[55]
18	Fitness Tracker	Yoga Pant	Wearable X	USA	US$250	[56]
19		Sport Bra	Supa	USA	US$100	[57]
20		T-shirt	Broadcastwear	India	US$45	[58]
21	Communication, Entertainment and Leisure	Jacket	Levi & Google	USA	US$350	[59]
22		Outerwear and underwear	Spinali Design	France	US$150~500	[60]

**Table 2:** Features of the textile components (TCs) of available SeCSs for health monitoring. ECG, electrocardiogram.

#	Product/Supplier	Textile Components (TCs)				Ref.
		Type	Key Design Features	Fabric Structure	Other Features	
1	Biodevices SA	Vest	Sleeveless	knitted (80% Polyamide, 20% elastane)	Disposable electrode	[37]
		T-shirt and baby bodysuit	Short sleeve			
2	Hexoskin	Vest	Sleeveless	knitted (73% micro polyamide, 27% elastane)	Anti-bacterial, UV protective, quick dry fabric	[38]
3	OM Signal	Shirt, Camisole	Short/long sleeve, sleeveless	knitted (blend of soft fibres)	Breathable and moisture management fabric, printed ECG sensor	[39]
		Bra	adjustable straps, removable and breathable padding, soft inner mesh, shock-absorbing racerback			
4	Emglare	Vest, T-Shirt	Sleeveless and short sleeve	knitted (100% recycled polyester)	Built-in heart rate monitor, ECG sensor, rechargeable lithium-ion battery, blue tooth antenna	[40]
		Bra and sport Bra	regular			
5	Healthwatch	Vest	Seamless knitting, front zipper. Sleeveless	knitted	Dry textile-electrodes, machine washable, with at least 50 washing cycles	[41]
6	Siren	Socks	Seamless knitting from yarn with embedded sensor	knitted	Machine washable and dry-able.	[42]
7	Neopenda	Baby hat	e-module attached to Knitted hat	knitted	Medical grade polymer and silicone	[44]
8	Mimo	Baby kimonos	baby onesie with two green stripes, a dock for the turtle module and snap button closure	knitted (single jersey, cotton)	Detachable e-module, washable	[45]
9	AiQ Bioman+	T-shirt	available as vest, shirt or sports bra	knitted	Conductive fibre-based textile electrodes, from stainless steel fibres	[47]
10	Myant Inc.	Underwear	underwear bottoms for both male and female, and sports bra	knitted	Sensors “knitted” into textiles	[48]
11	Smartlife	t-shirt, vest and bra	e-module attached at the front centre near under chest area	knitted	Detachable e-module	[49]
12	BioSerenity	Shirt and cap	Balaclava style knitted cap with integrated electrodes. Close-fitting short-sleeve t-shirt with sewn channels from conductive yarn	knitted	Detachable e-module for cap and t-shirt, washable	[51,61,62,63]

**Table 3:** Features of the electronics modules of available SeCSs for health monitoring. sEMG, surface electromyography; HR, heart rate; HRV, heart rate variability.

#	Supplier	E-Module			Sensing Parameters	Ref.
		Data Transfer	Assembly with Textiles	Compatible App/Software		
1	Biodevices SA	Bluetooth	Plug inside shirt pocket	VJ holter pro, Vitaljaket Telemetry	HR, HRV, ECG, movement	[37]
2	Hexoskin	Bluetooth	Plug inside vest pocket	Hexoskin, Hexoskin X, Apple Health App, Wear OS, MapMyRun, Runkeeper, Runtastic.	HR, HRV, HR recovery (HR2), breathing rate, step count, cadence, stride activity level, calories burned, sleep assessment	[38]
3	OM Signal	Bluetooth	Outer surface, on under chest	myHeart	ECG, respiration, physical activity	[39]
4	Emglare	Bluetooth	Integrated into clothing inside	Emglare Heart, Apple Heart, google Fit	Heart rate, ECG	[40]
5	Healthwatch	Wi-Fi, 3G, 4G	On outer surface above side left waist	Master caution	ECG, heart rate detection, skin temperature, respiratory, and body posture	[41]
6	Siren	Bluetooth	Above ankle	Siren App	Temperature	[42]
7	Neopanda	wireless	Around head	customised software	Pulse, respiratory rate, peripheral blood oxygen saturation, temperature	[43]
8	Mimo	Bluetooth to lillipad, Wi-Fi to app	Left side of stomach area	Mimo Monitor	Respiration, skin temperature, body position, activity level	[45]
9	AiQ	Bluetooth	Snaps onto garment, over left chest	-	Heart rate, respiration rate, skin temperature	[47]
10	Myant Inc.	Bluetooth	Slides into waistband	SKIIN	Heart rate, temperature, pressure, motion, body fat and hydration levels	[48]
11	Smartlife	Bluetooth	Placed in pocket of garment	-	Heart rate, respiration, Global Positioning System (GPS), ECG, and sEMG	[49]
12	BioSerenity	Bluetooth	Attached on top of head of cap and on the shoulders of t-shirt	Neuronaute app	EEG, EMG, ECG, and respiration rate and 9-axis accelerometer	[50,61,62,63]

**Table 4:** Features of the textile components (TCs) of available SeCSs for sports training.

Supplier	Textile Component (TC)				Ref.
	Type	Key Design Features	Fabric Structure	Other Features	
Athos	Men’s Shirt	Long sleeve, e-module snaps into the centre of front chest area	Knitted	sweat-wicking technology, compression	[51]
	Men’s Shorts	Elastic waist, e-module snaps into the side of thigh area			
	Women’s Legging	Elastic waist, e-module snaps into the side of thigh area			
Medtronic (Zephyr)	Compression T-shirt	Sleeveless, long length, centre chest e-module location, tight fit	Knitted (84% Polyester( PES), 16% Spandex)	conductive metallic fabric used as sensors, Stretchable	[52]
	Sports Bra	Securely sewn on back of bra with a neoprene backing to protect skin and e-module sensor, stretchable, medical-grade wiring connects sensors, e-module sensor on the back.	Knitted (88% PES, 12% Spandex)		
	loose fit shirt	Left side e-module location, semi-fitted athletic style, male and female fitting	Knitted	flame resistant, quick drying, machine washable at cold water (30 °C)	
Polar Team Pro	Sleeveless T-shirt	Fits fairly tight so that the electrodes are firmly against wearer’s skin	Knitted	machine washable at 40 °C/104 °F, No spin-dry, iron, dry clean, no bleach or softener	[53]
Komodotec	Compression Sleeve	sleeve with built in sensor on biceps and attachable device	Knitted	machine washable—100 times.	[54]
Sensoria	T-shirt	Short sleeve, tight fitting	Knitted (92% Polyamide, 8% elastane)	breathable, moisture wicking fabric, antimicrobial, machine washable	[55]
	Vest	E-module is attached with standard snaps at the centre of under chest, knitted wrinkle-pattern at front and back to ensure compression	Knitted (95% polyamide and 5% elastane)		
	Sports Bra	Elastic band, e-module to be attached with standard snaps at the centre of under chest	Knitted (74% polyamide, 18% polyester and 8% elastane)		
	Socks with anklet	Detachable anklet that snaps onto the sock	Knitted		

**Table 5:** Features of the electronics modules of available SeCSs for sports training.

#	Supplier	E-Module			Sensing Parameters	Ref.
		Data Transfer	Assembly with TC	Compatible App/Software		
1	Athos	Bluetooth	snaps into socket on Athos garments	Athos iOS app	sEMG, heart rate, calorie expenditure, and active time versus rest time.	[51]
2	Medtronic (Zephyr)	Bluetooth low energy and gateway	plugs into garment	OmniSense desktop software for windows	ECG, respiration, body temperature, accelerometery, time and location	[79,80]
3	Polar Team Pro	Bluetooth	slides into garment at centre back (CB) of neck	iPad app and web service	motion sensor, heart rate, GPS	[81]
4	Komodotec	Bluetooth	snaps into place at wrist	AIO sleeve app for android and apple	sleep analysis, health score, blood oxygen saturation level, ECG	[24]
5	Sensoria	Bluetooth Smart, ANT+	connect to snap buttons under chest	Sensoria Fitness App, compatible with 3rd party apps	heart rate	[25]
		Bluetooth	magnetically connect to socks, fold sock over anklet		cadence, foot landing and impact forces, step counting, speed, calories, altitude and distance tracking	

**Table 6:** Features of the textile components (TCs) of available SeCSs for fitness tracking.

Supplier	Textile Component (TC)				Ref.
	Type	Key Design Features	Fabric Structure	Other Features	
Sensoria	T-shirt, Vest	Short sleeve, tight fitting	Knitted (92% Polyamide, 8% elastane)	Breathable, moisture wicking fabric, antimicrobial, machine washable	[55]
	Sports Bra	E-module snaps at the centre of under chest, knitted wrinkle-pattern at front and back to ensure compression	Knitted (74% polyamide, 18% polyester, and 8% elastane)		
	Socks with anklet	Elastic band, e-module to be attached with standard snaps at the centre of under chest	Knitted		
Wearable X	Yoga Pant	E-module clips into the pants behind the left knee, flat seamed	Stretch, compression Knitted	Integrated sensors and haptic feedback (vibration) system, gentle wash and tumble dry.	[56]
Supa	Sport Bra	Classic racerback style finished with soft black elastic band	Knitted (95% PES, 5% Lycra)	Machine wash cold, hang dry and do not iron, do not bleach, remove the SUPA reactor before washing.	[57]
Syngal/Broadcast wearables	T-shirt	Short sleeve	Knitted	Soft switch, vibration sensors for navigating	[58]

**Table 7:** Features of the electronics modules of available SeCSs for fitness tracking.

Supplier	E-Module			Sensing Parameters	Ref.
	Data Transfer	Assembly with TC	Compatible App/Software		
Sensoria	Bluetooth Smart, ANT+	connect to snap buttons under chest magnetically connect to socks, fold sock over anklet	Sensoria Fitness App, compatible with 3rd party apps	heart rate cadence, foot landing and impact forces, step counting, speed, calories, altitude, and distance tracking	[55]
Wearable X	Bluetooth	clips into the host plate behind the left knee	Nadi X app	movement	[56]
Supa	Bluetooth smart	snaps to front under bust band	SUPA.AI app	heart rate	[57]
Syngal/Broadcast wearables	Bluetooth	sewn into garment	Syngal android app	GPS, calories, stairs climbed	[58]
